# *Ornithodoros sonrai* Soft Ticks and Associated Bacteria in Senegal

**DOI:** 10.3390/pathogens12091078

**Published:** 2023-08-24

**Authors:** El Hadji Ibrahima Ndiaye, Adama Zan Diarra, Fatou Samba Diouf, Charles Bouganali, Lionel Almeras, Cheikh Sokhna, Georges Diatta, Philippe Parola

**Affiliations:** 1 Faculté des Sciences Médicales et Paramédicales, Aix Marseille Univ IRD, AP-HM, SSA, VITROME, 19–21 Boulevard Jean Moulin, 13005 Marseille, France; el-hadji-ibrahima.ndiaye@ird.fr (E.H.I.N.); adamazandiarra@gmail.com (A.Z.D.); diouffatousamba@gmail.com (F.S.D.); almeras.lionel@gmail.com (L.A.); cheikh.sokhna@ird.fr (C.S.); 2 IHU Méditerranée Infection, 13005 Marseille, France; 3 VITROME, Campus International IRD-UCAD Hann, Dakar 1386, Senegal; charles.bouganali@ird.fr (C.B.); georges.diatta@ird.fr (G.D.); 4 Unité Parasitologie et Entomologie, Département Microbiologie et Maladies Infectieuses, Institut de Recherche Biomédicale des Armées, 13005 Marseille, France

**Keywords:** MALDI-TOF, *Ornithodoros sonrai*, *Borrelia*, *Coxiella burnetii*, *Bartonella*, Senegal

## Abstract

The soft ticks, *Ornithodoros sonrai*, are known as vectors of the tick-borne relapsing fever caused by *Borrelia* spp. and have also been reported to carry other micro-organisms. The objective of this study was to collect and to identify *O. sonrai* ticks and to investigate the micro-organisms associated with them. In 2019, an investigation of burrows within human dwellings was conducted in 17 villages in the Niakhar area and in 15 villages in the Sine-Saloum area in the Fatick region of Senegal. Ticks collected from the burrows were identified morphologically and by matrix-assisted laser desorption ionization time-of-flight mass spectrometry (MALDI-TOF MS). Micro-organism screening was performed by bacteria-specific qPCR and some identifications were made by standard PCR and gene sequencing. *O. sonrai* ticks were found in 100% (17/17) of the villages surveyed in the Niakhar area and in 66% (10/15) of the villages in the Sine-Saloum area. A total of 1275 soft tick specimens were collected from small mammal burrows. The ticks collected were morphologically identified as *O. sonrai*. About 20% (259/1275) of the specimens were also submitted to MALDI-TOF MS for identification. Among the resulting MS profiles, 87% (139/159) and 95% (95/100) were considered good quality specimens, preserved in alcohol and silica gel, respectively. All spectra of good quality were tested against our MALDI-TOF MS arthropod spectra database and identified as *O. sonrai* species, corroborating the morphological classification. The carriage of four micro-organisms was detected in the ticks with a high prevalence of *Bartonella* spp., Anaplasmataceae, and *Borrelia* spp. of 35, 28, and 26%, respectively, and low carriage of *Coxiella burnetii* (2%). This study highlights the level of tick infestation in domestic burrows, the inventory of pathogens associated with the *O. sonrai* tick, and the concern about the potential risk of tick involvement in the transmission of these pathogens in Senegal.

## 1. Introduction

Ticks are ectoparasitic acarids which are vectors of a large number of micro-organisms causing serious and potentially fatal diseases in animals, including humans [1]. There are approximately 870 species of ticks worldwide, divided into three families: the *Argasidae* or soft ticks, the *Ixodidae* or hard ticks, and the *Nuttalliellidae* [2,3,4]. Ticks have a worldwide distribution, although some species are geographically restricted [5,6]. There are over 170 species of *Argasidae* with non-sclerotised integuments (soft ticks). Within this family, *Ornithodoros sonrai* are known as the vector of the tick-borne relapsing fever caused by *Borrelia crocidurae* in most West African countries, including Senegal [5,7,8]. Rodents and insectivores are both hosts of *O. sonrai* and reservoirs of *B. crocidurae* [5]. Transovarial transmission of *B. crocidurae* in *O. sonrai* is also an important risk factor for the maintenance and spread of the pathogen [9].

Many burrows within human dwellings have been reported as being infested by *O. sonrai* in the northern two thirds of Senegal, with a prevalence ranging from 20 to 36% [5,8,10]. The prevalence of *B. crocidurae* carriage detected in *O. sonrai* ticks was found to be 13% in the Fatick region [8]. Clinical studies conducted in rural areas reported that tick-borne relapsing fever disease (TBRF) accounts for 5% of the reasons for medical consultation for febrile syndromes [11]. The incidence of borreliosis fluctuates from 4 to 26%, depending on the year and the area of Senegal [7,8,12,13,14,15]. One recent study which used an innovative approach involving the molecular analysis of malaria rapid negative diagnostic tests for *Plasmodium falciparum*, showed a high peak in the prevalence of infection due to the tick-borne borreliosis agent in July (16.47%) and August (11.21%) in health facilities in four regions of Senegal [16]. Apart from the carriage of *Borrelia*, few studies in Senegal have investigated whether the *O. sonrai* ticks harbour other micro-organisms [5,8,17,18,19,20]. *O. sonrai* ticks have also been reported to be carriers of *Coxiella burnetii*, the agent of Q fever in humans [17,21], bacteria from the *Bartonella* genus, and the bacterium *Occidentia massiliensis* gen. nov., sp. nov., a new member of the Rickettsiaceae family, the pathogenicity of which remains unknown [18,19,20,22].

To monitor ticks and control tick-borne diseases (TBDs), the accurate identification of the tick specimen at the species level and the determination of their infectious status are important [2]. MALDI-TOF MS has been shown to be an efficient tool for the identification of arthropods in general and ticks in particular [23,24,25,26].

In this study, we used MALDI-TOF to identify adult and nymphal stages of *O. sonrai* ticks collected in Senegal and preserved in alcohol and silica gel, and studied the carriage of tick-associated micro-organisms using molecular techniques.

## 2. Materials and Methods 

### 2.1. Study Sites and Tick Collection Periods

**Figure 1:**
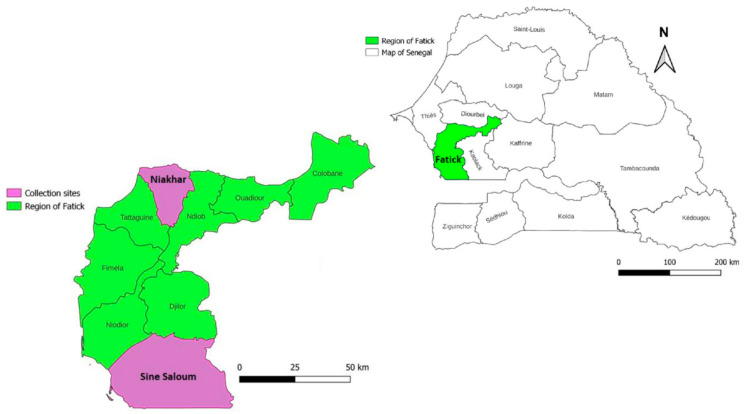
Map showing the geographic location of *O. sonrai* tick collection sites in rodent burrows in households in the Niakhar and Sine-Saloum area. The map was constructed using QGIS 3.28.0 software.

This study was conducted in two rural areas, the Niakhar area and the Sine-Saloum area (Dielmo/Ndiop and surrounding area) in the Fatick region in 2019. These areas have typical Sahelian and Sudano–Sahelian climates, respectively [27,28]. Rodent burrow surveys were conducted in 17 and 15 villages in the Niakhar and the Sine-Saloum areas, respectively (Figure 1).

Ticks were collected from burrows within domestic and peri-domestic homes. Verbal informed consent was obtained from the heads of the households prior to the collection of samples from rodent burrows located in the bedrooms, kitchens, stores and attics of human dwellings. Ticks were collected after asking residents a series of questions about their knowledge of arthropods and their potential presence in homes. Ticks were collected from between 15 and 30 small mammal burrows per village. The ticks were collected using a modified leaf vacuum cleaner, and samples were preserved in silica gel or 70% alcohol. Ticks from the same burrow were counted, pooled into a single tube, and stored at room temperature, before being sent to the VITROME laboratory (Marseille, France) and stored at +4 °C for analysis (import authorisation number ER23-2019). 

### 2.2. Morphological Identification of Ticks, Dissection and Sample Preparation 

Ticks were identified morphologically using previously established criteria [5,7,29,30]. Tick identification and determination of stage and/or sex were performed under a stereomicroscope at ×56 magnifications (Zeiss Axio Zoom.V16, Zeiss, Marly-le-Roi, France). Morphologically identified ticks were individually placed in 1.5 mL microcentrifuge tubes, rinsed once with distilled water and dried with paper. Each tick was dissected with a new sterile surgical blade to remove four legs for MALDI-TOF MS analysis and the body was cut lengthwise into two equal parts. The half of the body without legs was used for DNA extraction and molecular biology analysis and the other half was kept frozen (−20 °C) as a reserve for possible future testing [23].

### 2.3. DNA Extraction and Molecular Identification of Ticks 

Each half of the legless ticks was transferred to a 1.5 mL tube containing 180 μL of G2 lysis buffer and 20 μL of proteinase K (Qiagen, Hilden, Germany). It was then tissue-ground with metal beads and incubated at 56 °C overnight. DNA extraction was performed using an EZ1 DNA tissue kit (Qiagen) according to the manufacturer’s recommendations. DNA from each sample was eluted with 200 μL of Tris-EDTA (TE) buffer (Qiagen) and stored at −20 °C until use. Only specimens for which leg MS spectra were added to our reference spectra database were subjected to molecular identification. Their DNA was then subjected to standard PCR and sequencing in an automated DNA thermal cycler (Applied Biosystems, Foster City, CA, USA) using the 16S rDNA gene amplifying 420 base pair (bp) fragments (Table 1) [23,27,31]. DNA from laboratory-reared uninfected *Rhipicephalus sanguineus* s.l. ticks was extracted simultaneously with field-collected samples, and used as a negative control for standard PCR. PCR-positive amplicons were sequenced using the Big Dye Terminator Cycle Sequencing Kit (Perkin Elmer Applied Biosystems, Foster City, CA) and the resulting sequences were assembled using ChromasPro software (ChromasPro 1.7, Technelysium Pty Ltd., Tewantin, Australia). All assembled sequences obtained from standard PCR-amplified genes were compared to the NCBI GenBank (https://blast.ncbi.nlm.nih.gov/Blast.cgi accessed on 20 August 2023) by BLAST search to identify the closest genetically related genotypes. 

### 2.4. Sample Homogenisation for MALDI-TOF MS Analyses 

The four legs of the ticks were crushed with glass beads for three minutes in a mixture solution of 20 μL of 70% formic acid (Sigma, Lyon, France) and 20 μL of 50% acetonitrile (Fluka, Buchs, Switzerland) using the TissueLyser apparatus (QIAGEN, Germany) [27,37]. The homogenates were centrifuged and 1 μL of the supernatant was deposited in quadruplicate on a steel target plate (Bruker Daltonics, Bremen, Germany) [37]. After drying for a few minutes at room temperature, each spot was covered with 1 μL of matrix buffer composed of saturated α-cyano-4-hydroxycinnamic acid (CHCA, Sigma-Aldrich, Spruce Street, MO, USA), acetonitrile (50% v/v), trifluoroacetic acid (2.5% v/v) and high performance liquid chromatography (HPLC)-grade water. The plate containing the samples was then inserted into the MALDI-TOF MS instrument, as described previously [25]. *Escherichia coli* bacteria from our laboratory culture were deposited on each MS plate as a control.

### 2.5. MALDI-TOF Mass Spectrometry Parameters

Protein mass profiles were obtained using a MicroFlex LT MALDI-TOF Mass Spectrometer (Bruker Daltonics, Germany), with detection in the linear positive-ion mode at a laser frequency of 50 Hz within a mass range of 2–20 kDa. The setting parameters of the MALDI-TOF MS apparatus were identical to those previously used [34]. MS spectra were acquired automatically using the AutoXecute of the flexControl v.2.4 software (Bruker Daltonics).

### 2.6. MALDI-TOF Spectral Analysis, Database Creation

The MALDI-TOF MS profiles obtained from the four legs of the ticks preserved in silica gel or alcohol were evaluated by visually assessing the quality, intensity and reproducibility of all MS spectra obtained using flexAnalysis v.3.3 and ClinProTools 2.2 software (Bruker Daltonics). MS profiles judged to be of good quality are presented with high-intensity protein peaks (>3000 au), no background noise and a smooth baseline [27,38].

MS spectra which passed quality control criteria were selected for further analysis. MS profiles of samples preserved in alcohol and silica gel were compared with the MALDI-TOF MS dendrogram and principal component analysis (PCA) tools using MALDI Biotyper v3.0 and ClinProTools 2.2 software. To evaluate the ability of the MALDI-TOF MS tool to distinguish between male, female, and nymph ticks, MS spectra from randomly selected specimens were used for PCA, blind test and cluster analysis (dendrogram) using ClinProTools 2.2 and MALDI Biotyper v3.0, respectively. The performance of MALDI-TOF MS to distinguish ticks infected and not infected by *Borrelia* was also tested. Leg MS spectra from specimens whose infection status was confirmed by molecular biology were used to assess whether it is possible to separate these groups using PCA, comparison of discriminating peaks, and cluster analysis (dendrogram) using ClinProTools 2.2 and MALDI Biotyper v3.0, respectively.

The MS reference database (DB) was created using four and ten spectra of ticks preserved in silica gel and alcohol, respectively, using MALDI Biotyper (Bruker Daltonics). MS spectra were created with an unbiased algorithm using information on peak position, intensity and frequency. The reference spectra of 14 molecularly identified tick specimens used to create the database are available at the following link (https://doi.org/10.35081/zn4y-3s40 accessed on 20 August 2023). Our laboratory’s homemade MALDI-TOF MS arthropod database already contained the spectra of 169 arthropod species, including those of 46 tick species, as previously described [39,40].

### 2.7. MALDI-TOF Blind Test Identification

Blind tests were performed against the MS reference spectra DB update with the spectra of 220 remaining specimens, including 129 spectra from ticks preserved in alcohol and 91 from ticks preserved in silica gel. The reliability of species identification was estimated using log-score values (LSV) obtained from the MALDI Biotyper v.3.0 software, which ranged from 0 to 3 [27].

### 2.8. Molecular Detection of Tick-Associated Bacteria

Tick samples from which DNA was extracted were tested using quantitative real-time PCR (qPCR) for *Borrelia* spp., *Coxiella burnetii*, *Rickettsia* spp., *Bartonella* spp., the bacteria of Anaplasmataceae family, and *Wolbachia* spp. (Table 1) [32]. The reaction volume for real-time qPCR and reaction conditions has been described previously [8,41,42]. The qPCR amplification was performed using the CFX96 Real-Time system (Bio-Rad Laboratories, Foster City, CA, USA). Samples which had a cycle threshold (Ct) of below 36 were considered to be positive [8]. *Borrelia crocidurae* were researched by species-specific *glpQ* primers and probes, uniquely in specimens which were positive for *Borrelia* spp. by qPCR using the *ITS4* gene (Table 1). To consider a sample positive for *Coxiella burnetii*, both the *IS1111* and *IS30A* gene fragments must be detected by qPCR. DNA from *Rickettsia montanensis*, *Bartonella elizabethae*, *Anaplasma phagocytophilum*, *Coxiella burnetii*, *B. crocidurae*, and *Wolbachia pipientis* from our laboratory cultures were used as positive controls (Table 1) [32]. The preparation mixture (Roche mix (2×), sterile water without nucleotides, Uracil-DNA Glycosylase, primers and probe) without DNA was used for negative controls [8].

### 2.9. Standard PCR and Phylogenetic Analyses of Tick-Associated Bacteria

To confirm or determine bacterial species, qPCR-positive *Borrelia crocidurae*, *Bartonella* spp. and the bacteria of the Anaplasmataceae family were subjected to standard PCR. 

Ten qPCR-positive samples for *B. crocidurae* were sequenced by amplification of a 1360-bp fragment using the 16S *Borrelia* gene [33]. All *Bartonella* qPCR-positive samples were sequenced using the *gltA* and *ftsZ* genes amplifying 200-bp and 300-bp fragments, respectively [34,35]. All qPCR-positive samples from bacteria of the Anaplasmatacae family were first subjected to amplifying and sequencing of a 520-bp fragment of the *23S* rRNA gene [27]. In a second step, all samples with *23S* rRNA gene sequences close to *Ehrlichia* sp. were subjected to a second amplification of a 300-bp fragment of the *Ehrlichia* 1*6S* rRNA gene, and those close to *Wolbachia* were also subjected to amplification of a 500-bp fragment of the *Wolbachia* ftsZ gene [27,36]. The primers and probes used in this study are listed in Table 1.

The obtained sequences were assembled, corrected by Chromas Pro (Technelysium Pty. Ltd., Tewantin, Australia), and then compared with the sequences available in GenBank by BLAST search. The sequences of the *ftsz* and *gltA Bartonella* genes and the *23S* and *16S* genes of bacteria of the Anaplasmataceae family were used for phylogenetic analyses [27,34,35,36]. MEGA software version 7.0.21 was used to perform sequence alignments and construction of phylogenetic trees with 1000 bootstrap replications for the different genes. 

### 2.10. Statistical Analysis 

Statistical tests were used to compare the prevalence of tick infestations and micro-organism carriage in the study sites. The significance of explanatory variables and their interactions was determined by log-likelihood ratio tests. Means, standard deviations, and medians were calculated using the proportionality test. Tests for comparison of means were performed using the Kruskal–Wallis test with 95% confidence intervals, and a *p* value of <0.05 was considered to be statistically significant. All analyses were performed using R software v3.2.1.

## 3. Results

### 3.1. Tick Collection and Morphological Identification

All soft ticks collected from rodent burrows were morphologically identified under the stereomicroscope as *O. sonrai*. A total of 1275 *O. sonrai* tick specimens were collected, of which 615 specimens were preserved in silica gel and 660 in alcohol. Details about sampling sites and tick collection are available in Table 2 and Supplementary Tables S1 and S2. 

Among the sites investigated, *O. sonrai* ticks were found in 27/32 (84%) villages. These ectoparasites were detected in human dwellings in all villages in the Niakhar area (17/17) and in 10/15 (66%) villages inspected in the Sine-Saloum area (Figure 1, Table 2 and Supplementary Tables S1 and S2). *O. sonrai* ticks were found in 92/173 (53%) of the households inspected in all 17 villages of the Niakhar area and in 34/148 (23%) of the 10/15 villages in the Sine-Saloum area (Pearson chi-square test = 62.25; *p* = 0.0001). (Table 2 and Supplementary Tables S1 and S2). 

A total of 277/875 (32%) of the rodent burrows examined in the Fatick region were found to be infested with *O. sonrai* ticks. This prevalence was higher in the Niakhar area than in the Sine-Saloum area, representing 219/541 (40.5%) and 58/334 (17.3%) of burrow infestations, respectively, for the two localities (Pearson chi-square test, *p* = 0.0001) (Table 2 and Supplementary Tables S1 and S2). 

### 3.2. Molecular Identification of Ticks 

Twenty-two ticks were randomly selected for molecular identification. From them, the PCR products for the 16S-tick gene were obtained for 14 samples. The BLAST of the 16S gene sequences of the tick species revealed a strong and consistent identification of the species according to morphological identification, with coverage of between 99 and 100% and identity between 99.53 and 100% with *O. sonrai* sequences deposited in GenBank (GenBank accession numbers: OP947085 to OP947088).

### 3.3. MALDI-TOF MS Identification of O. sonrai Ticks

A total of 259 specimens of *O. sonrai*, comprising 72 males, 94 females and 93 nymphs, and randomly chosen from the specimens collected in all rodent burrows were subjected to MALDI-TOF MS for identification. A total of 100 specimens had been preserved in silica gel and 159 in alcohol (Table 3). Visualisation of the MS profiles using flexAnalysis v.3.3 software showed that 87.4% (139/159) and 95% (95/100) of the spectra obtained from the samples stored in alcohol and silica gel, respectively, met the criteria of sufficient quality for future analyses. The MS profiles obtained from the two preservation methods appeared to be different for all samples, as illustrated by the six representative specimens selected for each method, dendrogram and PCA analyses, which confirmed this difference between the MS protein peaks of *O. sonrai* tick specimens preserved in alcohol and silica gel (Supplementary Figure S1).

The MS spectra from the 14 specimens identified molecularly, including ten specimens preserved in alcohol and four in silica gel were added to our database. The blind test against the updated MALDI-TOF MS database revealed that all remaining spectra (n = 220) preserved in silica gel and alcohol matched with the *O. sonrai* species. The LSVs for specimens preserved in alcohol ranged from 1.6 to 2.4 (mean ± SD: 2.03 ± 0.17), while spectra preserved in silica gel had higher LSVs ranging from 1.8 to 2.6 (mean ± SD: 2.25 ± 0.17) (Table 3). The first identification hit corresponded to a specimen with the same preservation methods. 

**Figure 2:**
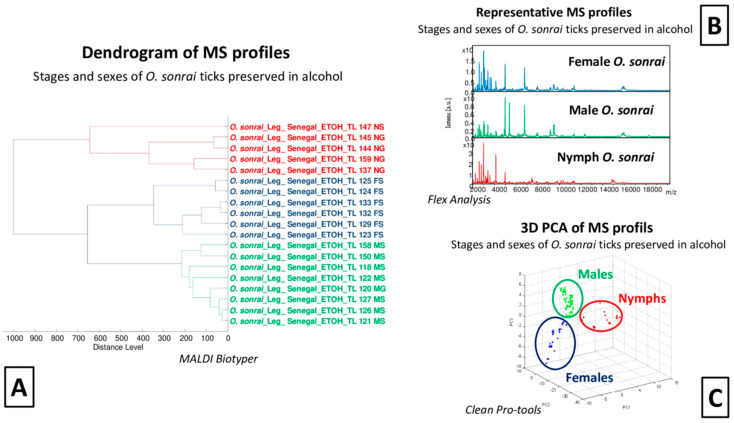
Comparison of MALDI-TOF MS spectra of *O. sonrai* ticks from the field preserved in alcohol. (**A**) MS dendrogram comparison of stage (adult and nymph) and sex (male and female), MS profiles using MALDI Biotyper software, version 3.0. (**B**) Representation and comparison of MS profiles of males (green), females (blue) and pupae (red) using flexControl 3.3 software. (**C**) PCA of MS spectra of males (green), females (blue) and pupae (red) using ClinProTools 2.2 software. a.u. = arbitrary units; m/z = mass to charge ratio; TL = TissueLyser, N = nymph; M = male; F = female; S = not-engorged, G = blood-engorged.

Visual comparison of MS spectra from ticks preserved in alcohol indicated that they seemed to be different, notably between stages of development (Figure 2B). The comparison of MS spectra using dendrogram analysis showed a clustering of protein profiles according to the stages separating nymphs from adult specimens but also distinguishing males from females at the adult stage (Figure 2A). The PCA performed on these same MS spectra confirmed a clear separation according to stage and gender for adult specimens (Figure 2C). The blind test correctly identified 95% of the stages or sex of the *O. sonrai* ticks preserved in alcohol. Conversely, for ticks preserved in silica gel, the dendrogram, PCA and blind tests were unsuccessful in distinguishing MS spectra according to stage or sex (Figure 2). 

The comparison of infected and uninfected MS protein peaks from 19 specimens preserved in alcohol (two non-engorged males, four non-engorged females, one engorged female, and 12 non-engorged nymphs) and ten specimens preserved in silica gel (three non-engorged males, three engorged males, one engorged female, and three engorged nymphs) encompassing engorging status, developmental stages, and different genera showed significant diversity in the MS profiles. MALDI-TOF tools failed to identify the *Borrelia* infection status of infected and uninfected ticks (see below), regardless of the method of preservation used, alcohol or silica gel (Supplementary Figure S2).

### 3.4. Molecular Detection of Micro-Organisms and Identification of Co-Infections 

DNA samples from 249 *O. sonrai* ticks were tested for the presence of bacteria using qPCR. The presence of DNA from *Borrelia* spp., *Coxiella burnetii*, *Bartonella* spp. and Anaplasmataceae families was detected in 26% (66/249), 0.8% (2/249), 35% (86/249), and 28% (69/249) of *O. sonrai* ticks tested by qPCR, respectively. *Rickettsia* spp. DNA was not detected in these *O. sonrai* tick samples (Table 4). The 66 *O. sonrai* ticks positive for *Borrelia* spp. were confirmed by specific qPCR to carry *B. crocidurae*. In the Niakhar and Sine-Saloum areas, *B. crocidurae* DNA was detected in 19% (23/119) and 33% (43/130) of the *O. sonrai* ticks tested, respectively. Carriage of *Bartonella* spp. DNA was detected in 51% (61/119) and 19% (25/130) of the ticks tested and Anaplasmataceae DNA was detected in 33% (39/119) and 23% (30/130) of the ticks from the Niakhar and Sine-Saloum areas, respectively. All *O. sonrai* ticks which were positive for *Coxiella burnetii* DNA (1.7%, 2/119) originated from the Niakhar area (Table 4). Fifty-five ticks presented co-infections, and two or three pathogenic agents were detected in 40 and 15 *O. sonrai* tick DNAs, respectively. The DNA of *B. crocidurae* and the bacteria of the Anaplasmataceae family was found in 16 ticks, those of *B. crocidurae* and *Bartonella* spp. in ten, and finally those of *B. crocidurae* and *Coxiella burnetii* in one specimen. The prevalence of double carriage was obtained from non-*Borrelia* DNA in ticks between the Anaplasmataceae family and *Bartonella* spp. for 15 ticks. DNA of *B. crocidurae*, the Anaplasmataceae family and *Bartonella* spp. was found in 14 ticks and that of *B. crocidurae*, the Anaplasmataceae family and *Coxiella burnetii* in one *O. sonrai* tick (Table 4).

### 3.5. Standard PCR and Phylogenetic Analyses of Micro-Organisms

The PCR products of 4/10 *Borrelia*-positive randomly selected tick DNA samples were obtained and sequenced. The BLAST analysis of 1360 bp sequences of the 16S_Bor gene of the four specimens showed that they were between 99.70 and 100% identical to the sequence of *B. crocidurae* identified in Mauritania (GU350713), Morocco (DQ057990) and Senegal (U42285).

**Figure 3:**
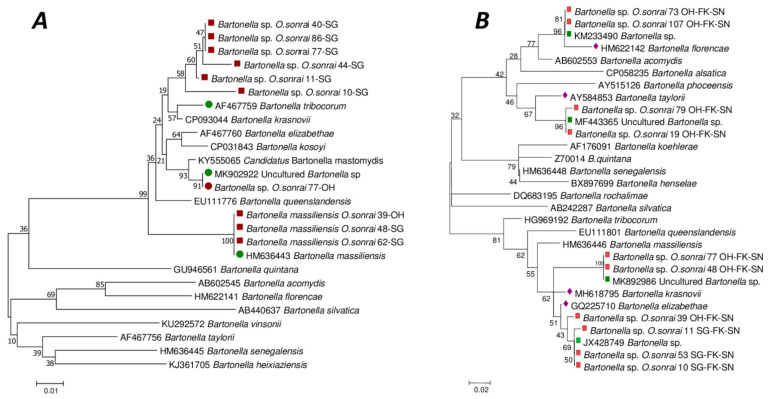
Phylogenetic tree (neighbourhood joining, 1000 bootstrap replicates) based on *Batonella* partial sequences of the cell division protein gene Ftsz (263 bp) (**A**) and the citrate synthase gene gltA (173 bp) (**B**), showing the relationships of the *Bartonella* species studied. Red square = our Bartonella sequence from *O. sonrai*; Green circle and Green square = Genbank identification first hits; Purple diamond = most closely related known species.

*Bartonella* sequences were obtained from 36/86 (42%) and 13/86 (15.12%) samples that were positive by qPCR to *Bartonella* spp. for the *gltA* and *ftsZ* targets, respectively. The 200 bp *gltA* gene sequences obtained identified four different undescribed *Bartonella* species. Meanwhile, the 300 bp *ftsZ* amplicon sequences confirmed the presence of two known *Bartonella* species, and one as yet undescribed *Bartonella* species. Comparison of our 36 *gltA* gene sequences with the GenBank database indicated that 27/36 (75%) were similar to a *Bartonella* sp. (JX428749) detected in a rodent, *Cricetomys gambianus*, with genetic identities ranging from 98.49 to 100%. The closest known species to these 27 sequences was *B. elizabethae* (GenBank accession number: GQ225710), with genetic similarities ranging from 98.45 to 98.98%. In addition, 4/36 (11%) were 100% similar to a *Bartonella* sp. (GenBank accession number: KM233490) detected in the shrew *Crocidura olivieri*. The closest known species to these four sequences was *B. florencae* (GenBank accession number: HM622142) with a genetic similarity of 97.46%. Meanwhile, 3/36 (8%) were 99.50 and 100% identical to an uncultured *Bartonella* sp. (GenBank accession number: MF443365) detected in *Xenopsylla cheopis*. The closest known species to these three sequences was *B. taylorii* (GenBank accession number: AY584853) with a genetic similarity of 94.47%. Finally, 2/36 (6%) sequences were 100% identical to an uncultured *Bartonella* sp. (GenBank accession number: MK892986) and close to *B. elizabethae* (GenBank accession number: MK660539), with a genetic identity of 98.49% (Supplementary Table S3). Of the 13 reliable sequences obtained from *ftsZ* amplicons, 9/13 (69%) were close to *B. tribocorum* (identity ranging from 97.25 to 97.60%) isolated from *Rattus norvegicus* rats in France (GenBank accession number: AF467759) and 3/13 (23%) were identified as 100% identical to *B. massiliensis* (GenBank accession number: HM636443) isolated from the *O. sonrai* tick in Senegal. In addition, 1/13 (8%) sequences were 100% identical to an uncultured *Bartonella* sp. (GenBank accession number: MK902922) detected in *Mastomys erythroleucus* rodents in Mali, and close to *B. Mastomydis* (GenBank accession number: KY555065), with a genetic identity of 98.97% (Supplementary Table S3). The phylogenetic position of *Bartonella* detected in our study with the *ftsZ* and *gltA* genes are shown in the phylogenetic trees in Figure 3A and Figure 3B, respectively. Our *Bartonella ftsZ* and *gltA* genes sequences have been deposited in GenBank under accession numbers OP947080 to OP947084 and OP948207 to OP948216, respectively.

**Figure 4:**
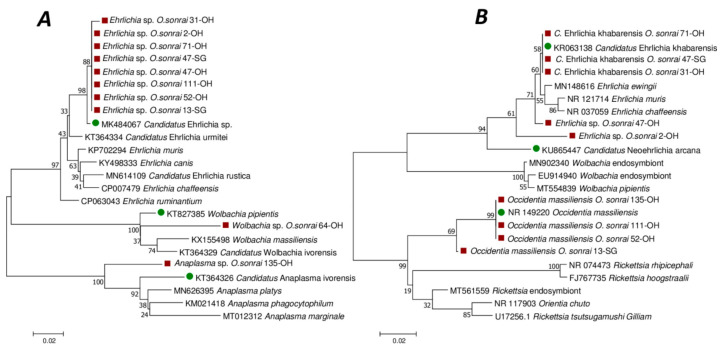
Phylogenetic tree (neighbour joining, 1000 bootstrap replicates) based on partial sequences of the Anaplasmataceae 23S (434 bp) (**A**) and 16S (278 bp) ribosomal RNA genes (**B**), showing the relationships between the species of the Anaplasmataceae family studied. Red square = our Anaplasmataceae sequence from *O. sonrai*; Green circle = Genbank identification first hits.

For the qPCR-positive samples from bacteria of the Anaplasmataceae family, 38/69 (55.07%) sequences were obtained with the *23S* rRNA gene. The BLAST of these sequences showed that they belonged to three different genera, including *Ehrlichia*, *Wolbachia* and *Anaplasma* (Figure 4A). Thirty-six sequences were close to the *Ehrlichia* genus and had 98.76 to 99.36% identity with the corresponding sequence of *Candidatus* Ehrlichia sp. isolated from the spleen of a rodent in Senegal (GenBank accession number: MK484069). Our *Ehrlichia*, *Wolbachia* and *Anaplasma 23S* gene sequences were deposited in GenBank under accession numbers OP935908 to OP935910, OP935907 and OP935906 respectively.

Sequencing these 38 samples with the 16S *Ehrlichia* gene yielded 36 sequences. BLAST of these sequences showed that 28 had 97–100% identity with the *Occidentia massiliensis* sequence (with GenBank accession number: NR149220), while 5/36 had 95–100% identity with an uncultured *Ehrlichia* sp. (GenBank accession number: MH250197), and three had 99% identity with *Candidatus* Ehrlichia khabarensis (with GenBank accession number: KR063138). Our *Occidentia 16S* gene sequences were deposited in GenBank under accession numbers OP947012 to OP947019.

The phylogenetic position of the sequences obtained with the *16S Ehrlichia* gene are represented on the phylogenetic tree in Figure 4B.

Only one 23S rRNA presented 99.13% similarity with the corresponding sequence of *Wolbachia* endosymbiont detected from *Ctenocephalides felis* in California (GenBank accession number: CP051156). BLAST of the sequence obtained for this sample with the *Wolbachia*-specific *ftsZ* gene showed 93% identity with *Wolbachia* sp. detected on *Cimex* japonicus in Japan (GenBank accession number: AB508953). The last 23S rRNA sequence had 94% identity with uncultured *Anaplasma* sp. detected in the spleen of *Mastomys natalensis* in Tanzania (GenBank accession number: OL982744).

## 4. Discussion

The present study reports a high level of infestation (40%) with *O. sonrai* ticks in burrows within houses in the Niakhar area. The Dielmo/Ndiop and surrounding areas had a lower infestation level of 17.3% in the burrows of domestic and peri-domestic dwellings. The levels of *O. sonrai* tick infestations obtained in the burrows of the two localities were higher than those reported in previous studies conducted in these same localities [7,8]. A high prevalence of 36.4% was reported in domestic and peri-domestic dwellings in the Niakhar area in 2016 and 17% in the Dielmo/Ndiop area in 2006 [7,8]. The Niakhar area was reported to be heavily infested with the *O. sonrai* tick in burrows within homes [8]. Meanwhile, the villages of Dielmo and Ndiop and their surroundings area were reported as marking the southern limit of the vector’s distribution [5,7,43]. However, in the villages of Dielmo and Ndiop, an alternative control strategy was developed between 2013 and 2015 with the objective of improving intra-household housing conditions by cementing all rooms to avoid the risk and frequency of human contact with ticks [44]. This measure significantly reduced the numbers of infested burrows and the transmission of relapsing fever and is likely to have been responsible for the very low prevalence of tick infestation in burrows within homes observed in these two villages.

The presence of the *O. sonrai* tick vector has been reported in all countries of West and North Africa and its distribution is well-established [5,45]. The limits of the geographical distribution as well as the climatic and geographical parameters involved in the distribution of the *O. sonrai* soft tick have been well known since 2013 [5,28,45]. The *O. sonrai* tick is specifically found in the burrows of small mammals and insectivores in the wild, and is found in dwellings when burrows open within them [7,8,32]. A high level of burrow infestation has been reported in Senegal with variable prevalence depending on localities and rainfall [8,43,45], confirming that the site is an important focus of TBRF in Senegal [5,8,45]. 

The *O. sonrai* tick specimens collected in this study were identified morphologically and some species were confirmed by molecular biology. The MALDI-TOF MS tool was applied as an innovative approach towards tick identification by proteomics to confirm the morphological identification. MALDI-TOF MS is a fast and modern tool which is rapidly expanding in the field of medical entomology, enabling the accurate identification of arthropods as well as, in certain circumstances, their sex, geographical origins, blood meals and even associated bacteria [25,27,37,46]. In this study, the dissection of the legs of each tick, as already described [23,37], was used to identify the specimens at the species level during our blind tests. Given the large number of *Ornithodoros* ticks in Africa and the poor morphological criteria for differentiating species from this genera [5,47], accurate and rapid identification using MALDI-TOF MS seems to be an important opportunity to consolidate the morphological identification criteria of the species. 

In this study, two methods of preserving field tick specimens (alcohol and silica gel) were compared for better MALDI-TOF MS analysis performance. MALDI-TOF MS profiles of *O. sonrai* tick specimens preserved in silica gel produced a greater proportion (95%) of good quality spectra compared to those from specimens preserved in alcohol (87.4%). The results obtained with both storage methods were fairly good, probably because they were not stored long enough (less than one year) to undergo degradation. Sample storage time is a very important factor in MALDI-TOF analysis because it can cause protein degradation, leading to the adaptation of study protocols to obtain good quality MS spectra [40]. Studies have reported the effect of the continuous alteration of MS profiles on samples stored in alcohol for several years [25,48]. The MALDI-TOF tool was able to correctly identify 100% of the specimens according to their preservation mode (silica gel and alcohol). PCA analysis, dendrogram and blind test identification of the MS spectra of tick specimens preserved in alcohol allowed us to differentiate between nymphs and adults, and between male and female genera collected in the field. Our results show that there is a difference between the MS spectra of males, females, and nymphs of our alcohol-preserved soft tick specimens, which previous studies did not obtain from the legs of two hard tick species preserved in 70% ethanol [49]. However, our results are comparable to another study performed on the legs of four mosquito species, which clearly demonstrated the sex difference in MALDI-TOF MS profiles [46]. The preservation of arthropods in silica gel seems to offer more reliability and greater opportunities for MALDI-TOF MS analysis for species, stage, and sex identification, as previously demonstrated [27,46]. But, this was not the case in our study. The tested tick leg samples, preserved in silica gel, did not enable us to discriminate between nymphs and adults nor between adult male and female genera. However, recent studies have shown that storing samples in alcohol for a long period of time causes denaturation and the alteration of protein compounds affecting the efficiency of MALDI-TOF MS analyses and MS profiles [48]. The sensitivity of MALDI-TOF MS analysis can induce variation in the proteins obtained due to the sampling procedure, storage of biological material, and dissection of each sample, hence the need for rigour and precision at each step [27]. A previous study reported that MALDI-TOF MS was capable of differentiating *O. sonrai* ticks infected with *B. crocidurae* [37] from those which were not. In our study, we were unable to distinguish the MS leg spectra from *Borrelia*-infected and non-*Borrelia*-infected ticks. Several factors could explain this discrepancy. Firstly, in the present study, ticks were stored using two storage media, alcohol or silica gel, whereas fresh *O. sonrai* tick specimens were used in the previous study. Here, and in previous studies, it was repeatedly reported that the storage medium and its duration can significantly alter the MS spectra and the direct comparison of spectra from specimens of the same species but stored in different conditions is not then possible [25,27,40,48]. Additionally, it has been demonstrated that the choice of body part, blood-engorged status, and developmental stage, as well as sample processing method and the quality of dissection have a significant impact on the resulting MS spectra [25,27]. Our MS profiles showed a significant variability in protein peaks that may be induced by stages (adults and nymphs), genera (males and females), engorgement status (blood-engorged and non-blood-engorged ticks), and infection status for the multiple pathogens detected, which could explain our inability to identify infection status in our analyses. 

*O. sonrai* ticks are vectors of TBRF and use as their main reservoir of hosts small mammals which may carry various micro-organisms, including pathogens [8,43,50,51]. In this study, we detected a total of six genera of bacteria (*Borrelia* spp., *Bartonella* spp., *Anaplasma* spp., *Coxiella burnetii*, *Ehrlichia* spp. and *Occidentia* spp.) and one endosymbiont bacterium (*Wolbachia* spp.) using standard qPCR and PCR tools. We identified high species diversity in the bacteria of the genera *Bartonella* spp. and the family Anaplasmataceae by phylogeny-confirmed BLASTN sequences, some of which are known and isolated, and others which have not yet been described (Figure 3 and Figure 4). In this study, we report, for the first time in *O. sonrai,* the carriage of endosymbiont *Wolbachia* spp. The carriage of *B. crocidurae*, *B. massiliensis*, *Coxiella burnetii*, *Occidentia massiliensis, Anaplasma* spp., *Candidatus Ehrlichia* spp., and *Bartonella* sp. had been reported by previous studies [17,19,20,22,45,52].

*Bartonella* spp. are Gram-negative, haemotropic, aerobic and non-motile bacteria, transmitted mainly by arthropod vectors [18,34]. *Bartonella* are responsible for human diseases, the best known of which are Carrión disease, trench fever and cat scratch disease. *Bartonella* species are also associated with chronic bacteraemia and/or endocarditis, bacillary angiomatosis, peliosis hepatis, prolonged fever of unknown origin, retinitis, uveitis and myocarditis in humans [53]. In Senegal, *Bartonella* spp. have already been detected in *O. sonrai* ticks with a prevalence of (5/8, 62.5%) and (1/24, 4.2%) in two villages, Soulkhou Thisse and Maka Gouye, respectively. This *Bartonella* has been described as a new species named *Bartonella massiliensis*, which was also identified in the present study [18,19]. However, in *O. sonrai* ticks from Senegal, the carriage of other undescribed *Bartonella* species was reported. These *Bartonella* spp. are identical and/or closely related to uncultured *Bartonella* spp. identified in rodents and insectivores from Mali (MK902922, MK892986), Uganda (MF443365, JX428749) and Kenya (KM233490).

*Coxiella burnetii*, the agent of Q fever, can cause non-specific febrile illness, pneumonia, hepatitis, endocarditis and vascular infection in humans [54]. A strict gram-negative intracellular bacterium, *C. burnetii* infects many animals, arthropods and humans. Ticks can also serve as vectors [23,32]. However, the main source of infection is aerosols or direct contact with infected ruminants and their products. Although ticks are competent vectors for *C. burnetii* in experimental models, only a few cases of Q fever caused by tick bites have been reported [21]. A recent study reported a human case of acute Q fever caused by infected *Rh. pusillus* ticks from southern France [6]. Here, *C. burnetii* DNA was detected in 2% of *O. sonrai* ticks only in the Niakhar area. Another study conducted in Senegal reported the carriage of *C. burnetii* in the *O. sonrai* tick in Maka Gouy in the Fatick region, located at the same latitude as the Dielmo/Ndiop area at 159 km away as the crow flies [17]. 

The Anaplasmataceae family includes bacteria of the genera *Anaplasma*, *Ehrlichia*, *Neorickettsia* and *Wolbachia*, which are Gram-negative intracellular organisms [55]. The prevalence of these bacteria in ticks varies from country to country and from tick species to species [56]. Herein, DNA from the Anaplasmataceae family was detected in 28% of *O. sonrai* ticks. *Anaplasma* sp. and *Ehrlichia* sp. have already been detected in these *Ornithodoros* ticks at Dodel in the Saint-Louis region of Senegal. However the *Anaplasma* sp. and *Ehrlichia* sp. were different from those detected in this study [52]. Our *Anaplasma* sp. *and Ehrlichia* sp. are closer to those detected in rodents and shrews in Gabon (MT269273) and Russia (KR063138), respectively. This work reports, for the first time, the presence of *Wolbachia* sp. in *O. sonrai* ticks collected from small mammals’ burrows in Senegal.

## 5. Conclusions

This study shows the importance of the conservation and status of the samples for MALDI-TOF analyses which, despite the price of the equipment, can be used in several disciplines such as entomology, bacteriology, virology, parasitology and mycology. Running costs, other than maintenance, are very low [46,57]. Our MALDI-TOF MS results demonstrate the epidemiological importance of identifying arthropods for better management. It would be interesting to develop a MALDI-TOF MS protocol capable of identifying both ticks and the multiple microorganisms they may harbour. Furthermore, a significant number and percentage of known and potentially new pathogenic species were identified in the *O. sonrai* ticks, which are increasingly becoming a public health threat. DNA from *B. crocidurae*, *C. burnetii*, *O. massiliensis*, *Wolbachia endosymbiont*, *Candidatus Ehrlichia khabarensis*, *Ehrlichia* sp., *Anaplasma* spp., *B. massiliensis* and *Bartonella* sp. were detected in *O. sonrai* soft ticks. Further studies are needed to culture and isolate these detected pathogens in order to properly describe potential new species. This study paves the way for experimental testing of the transmission of these bacteria carried by the O. sonrai ticks.

## Figures and Tables

**Table 1:** Primers and probes used in this study for real-time and standard PCR.

System Name	Assay Specificity	Targeted Gene	Primer Sequences	Reference
Real Time Quantitative PCR	*Borrelia* spp.	*ITS4*	Bor_ITS4_ F (5’-GGCTTCGGGTCTACCACATCTA-3’) Bor_ITS4_ R (5’-CCGGGAGGGGAGTGAAATAG-3’) Bor_ITS4_ P (6FAM-TGCAAAAGGCACGCCATCACC)	[32]
	*B. crocidurae*	*GlpQ*	Bcroci_glpQ_F (5’-CCTTGGATACCCCAAATCATC-3’) Bcroci_glpQ_R (5’-GGCAATGCATCAATTCTAAAC-3’) Bcroci_glpQ_P (6FAM-ATGGACAAATGACAGGTCTTAC –MGB	
	*Bartonella* spp.	*ITS2*	Barto_ITS2_F (5’- GGGGCCGTAGCTCAGCTG-3’) Barto_ITS2_R (5’- TGAATATATCTTCTCTTCACAATTTC-3’) Barto_ITS2_P (6FAM- CGATCCCGTCCGGCTCCACCA)	[32]
	Anaplasmataceae	*qANA*	TtAna_F (5’-TGACAGCGTACCTTTTGCAT-3’) TtAna_R (5’- GTAACAGGTTCGGTCCTCCA-3’) TtAna_P (6FAM- GGATTAGACCCGAAACCAAG)	[32]
	*Coxiella burnetii*	*ISS11*	CB_IS1111_0706F (5’-CAAGAAACGTATCGCTGTGGC-3’) CB_IS1111_0706R (5’-CACAGAGCCACCGTATGAATC-3’) CB_IS1111_0706P (6FAM-CCGAGTTCGAAACAATGAGGGCTG)	[32]
		*IS30A*	CB_IS30A_3F (5’- CGCTGACCTACAGAAATATGTCC-3’) CB_IS30A_3R (5’-GGGGTAAGTAAATAATACCTTCTGG-3’) CB_IS30A_3P (6FAM- CATGAAGCGATTTATCAATACGTGTATGC)	[32]
	*Rickettsia*	*RKND*	RKND03_F (5’-GTGAATGAAAGATTACACTATTTAT-3’) RKND03_R (5’-GTATCTTAGCAATCATTCTAATAGC-3’) RKND03_R (6FAM- CTATTATGCTTGCGGCTGTCGGTTC)	[32]
Standard PCR	Ticks	*16S*	16S_Tick_F (5′-CCGGTCTGAACTCAGATCAAGT -3′) 16S_Tick_R (5′-GCTCAATGATTTTTTAAATTGCTGT-3′)	[23]
	*Borrelia* spp.	*16S*	B_F1 (5’-GCTGGCAGTGCGTCTTAAGC -3’) B_R1 (5’-GCTTCGGGTATCCTCAACTC -3’)	[33]
	*Anaplasmataceae*	*23S rRNA (rrl)*	Ana23S-212_F (5’-ATAAGCTGCGGGGAATTGTC-3’) Ana23S-753_R (5’-TGCAAAAGGTACGCTGTCAC-3’)	[27]
	*Bartonella* spp.	*FtsZ*	FTSZDIR-F (5’-CCGTGAATAATATGATTAATGC-3’) FTSZREV-R (5’-TTGAAATGGCTTTGTCACAAC-3’)	[34]
		*GltA*	GltA-F (5’- TTACTTATGATCCKGGYTTTA-3’) GltA-R (5’-AATGCAAAAAGAACAGTAAACA-3’)	[35]
	*Wolbachia* spp.	*FtsZ*	Wol-99F (5’-TTGTAGCCTGCTATGGTATAACT-3’) Wol-994R (5’-GAATAGGTATGATTTTCATGT-3’)	[27]
	*Ehrlichia* spp.	*16S*	Ehr-16S-F (5’-GGTACCYACAGAAGAAGTCC-3’) Ehr-16S-R (5’-TAGCACTCATCGTTTACAGC-3’)	[36]

**Table 2:** Intra and peri-residential prospection of rodent burrows in the Niakhar and Sine-Saloum areas.

Area of Collection	Villages Infested/Prospected	Households Investigated	Positive Households (%)	Burrows Prospected	Positive Burrows (%)	Tick Storing Medium	Number of Specimens Collected
Niakhar	17/17	173	92 (53%)	541	219 (41%)	Alcohol	338
						Silica gel	481
Sine-Saloum	10/15	148	34 (23%)	334	58 (17%)	Alcohol	322
						Silica gel	134
Total	32	321	126 (39%)	875	277 (32%)	1275	

**Table 3:** Number of *O. sonrai* tick specimens tested according to sample preservation method for MALDI-TOF MS analysis, creation of the reference database and blind test results for MS identification and log-score values of each species.

Storage Medium	Area	Number of Villages	% MALDI-TOF MS Specimens Tested/Collected	% Good Spectrum	Spectra in Data	% Identification [LSVs Range]
Alcohol	Niakhar	2/17	10/338	50% (5/10)	1	100% (4/4) [1.8–2.5]
	Sine-Saloum	6/15	149/322	89% (134/149)	9	100% (125/125) [1.6–2.5]
Silica gel	Niakhar	9/17	100/481	95% (95/100)	4	100% (91/91) [1.8–2.6]
Total		17/32	23% (259/1141)	90% (234/259)	14	100% (220/220) [1.6–2.6]

**Table 4:** Number of *O. sonrai* tick specimens tested positive for various bacteria by qPCR.

*qPCR* Pathogen Tested	Proportion of Positive Samples Per Village		Total
	Sine-Saloum	Niakhar	
Prevalence of pathogen detected in *O. sonrai*			
*Borrelia crocidurae*	33%; 43/130	19%; 23/119	26%, 66/249
*Coxiella burnetii*	0%; 0/130	1.7%; 2/119	0.8%, 2/249
*Bartonella* spp.	19%; 25/130	51%; 61/119	35%, 86/249
*Anaplasmataceae*	23%; 30/130	33%; 39/119	28%, 69/249
*Rickettsia* spp.	0%; 0/130	0%; 0/119	0%
Prevalence of double co-infections detected			
*B. crocidurae + Anaplasmataceae*	8.5%; 11/130	4.2%; 5/119	6.4%, 16/249
*B. crocidurae + Bartonella* spp.	6.2%; 8/130	1.7%; 2/119	4%, 10/249
*B. crocidurae + C. burnetii*	0%; 0/130	0.8%; 1/119	0.4%, 1/249
*Anaplasmataceae + Bartonella* spp.	2.3%; 3/130	10%; 12/119	6%, 15/249
Prevalence of triple co-infections detected			
*Anaplasmataceae + B. crocidurae + Bartonella* spp.	4.6%; 6/130	6.7%; 8/119	5.6%, 14/249
*Anaplasmataceae + B. crocidurae + C. burnetii*	0%; 0/130	0.8%; 1/119	0.4%, 1/249

## Data Availability

All relevant data are presented in the paper and in the accompanying Supplementary Data. Our MALDI-TOF MS database is publicly accessible on our laboratory website and can be downloaded with the following DOI number: https://doi.org/10.35081/zn4y-3s40. More data presented in this study are available on request from the corresponding author.

## Supplementary Materials

The following supporting information can be downloaded at: https://www.mdpi.com/article/10.3390/pathogens12091078/s1, Table S1: Intra and peri-residential prospection of rodent burrows in the Niakhar area; Table S2: Intra and peri-residential prospection of rodent burrows in the Sine-Saloum area; Table S3: Identification of *Bartonella* spp. with the closest known species using two different genes from *O. sonrai* ticks in Senegal. Figure S1: Comparison of MALDI-TOF MS spectra of *O. sonrai* ticks from the field preserved in silica gel (red) and alcohol (green); (A) representation and comparison of MS profiles using FlexControl 3.3 software. (B) Dendrogram representation of variability between MS profiles using MALDI Biotyper software, version 3.0; (C) PCA of MS profiles using ClinProTools 2.2 software. a.u. = arbitrary units; m/z = mass to charge ratio; TL = TissueLyser; Figure S2: Comparison of MALDI-TOF MS spectra of Borrelia-infected (red) and uninfected (green) O. sonrai ticks from the field preserved in silica gel (A1–3) and alcohol (B1–3). Dendrogram representation of the variability between MS profiles preserved in silica gel (A1) and alcohol (B1) using MALDI Biotyper software, version 3.0. Representation and comparison of the average MS profile preserved in silica gel (A2) and alcohol (B2) using ClinProTools 2.2 software. PCA of the MS spectra preserved in silica gel (A3) and alcohol (B3) using ClinProTools 2.2 software. a.u. = arbitrary units; m/z = mass/charge ratio; TL = TissueLyser, N = nymph; M = male; F = female; S = not-engorged, G = blood-engorged.
