# Supplementary material for: Ornithodoros sonrai Soft Ticks and Associated Bacteria in Senegal
Source: Pathogens. 2023 Aug 24;12(9):1078. doi: 10.3390/pathogens12091078 (PMC10534570; doi:10.3390/pathogens12091078)
Supplement: Supplementary file 1 [file pathogens-12-01078-s001.zip › pathogens-2494957-supplementary.pdf]

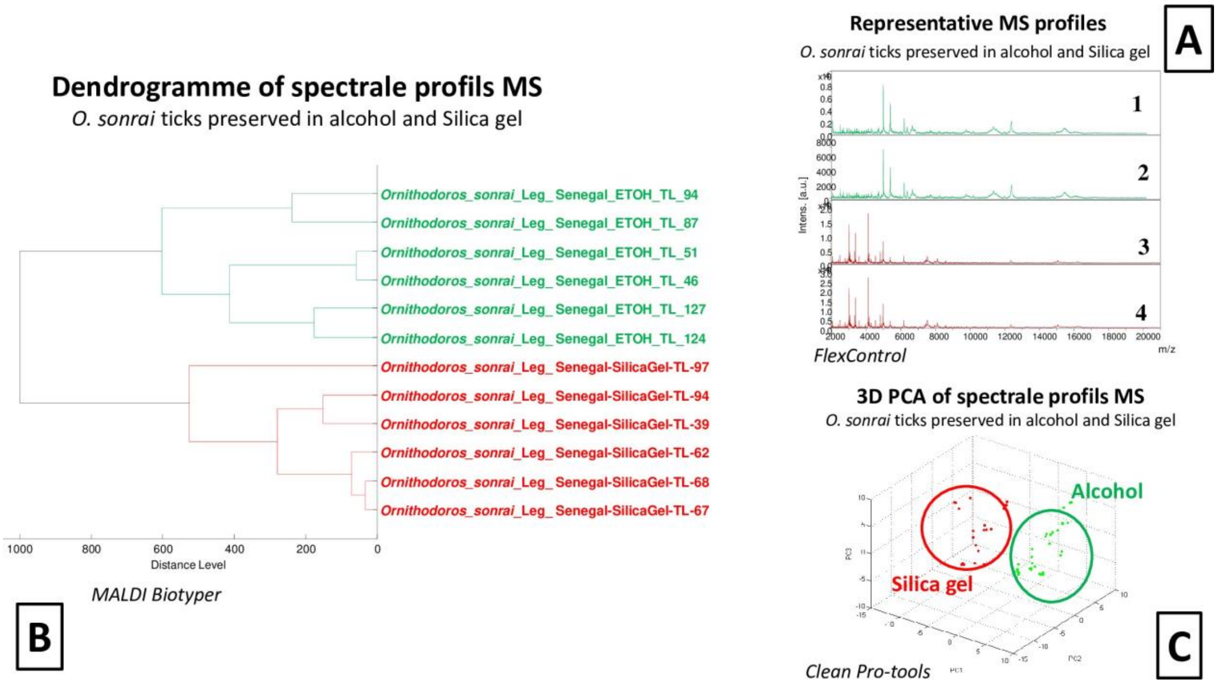

Supplementary Figure S1. Comparison of MALDI-TOF MS spectra of *O. sonrai* ticks from the field preserved in silica gel (red) and alcohol (green); (A) Representation and comparison of MS profiles using FlexControl 3.3 software. (B) Dendrogram representation of variability between MS profiles using MALDI Biotyper software, version 3.0; (C) PCA of MS profiles using ClinProTools 2.2 software. a.au = arbitrary units; m/z = mass to charge ratio; TL= TissueLyser.

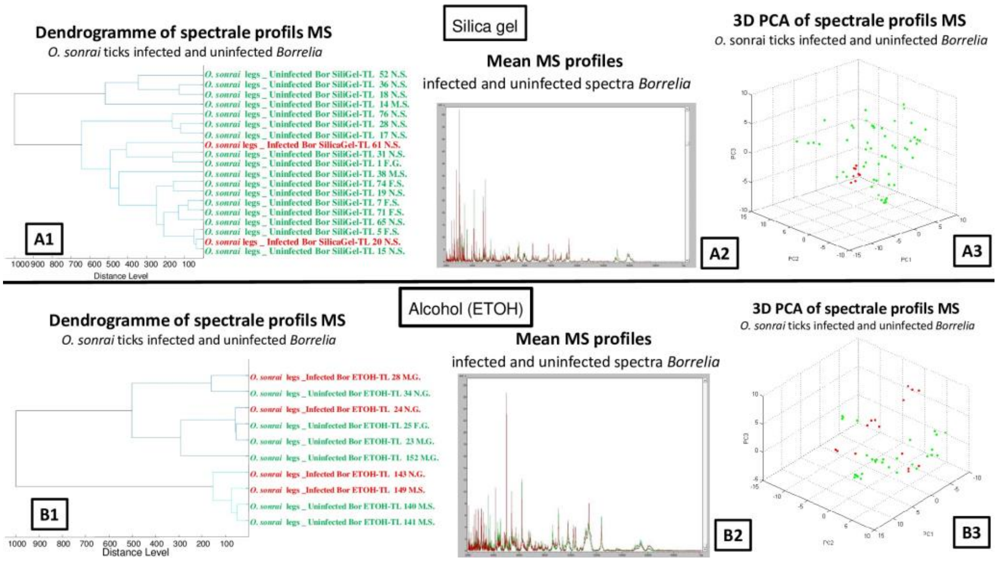

Supplementary Figure S2. Comparison of MALDI-TOF MS spectra of Borrelia-infected (red) and uninfected (green) O. sonrai ticks from the field preserved in silica gel (A1-3) and alcohol (B1-3). Dendrogram representation of the variability between MS profiles preserved in silica gel (A1) and alcohol (B1) using MALDI Biotyper software, version 3.0. Representation and comparison of the average MS profile preserved in silica gel (A2) and alcohol (B2) using ClinProTools 2.2 software. PCA of the MS spectra preserved in silica gel (A3) and alcohol (B3) using ClinProTools 2.2 software. a.au = arbitrary units; m/z = mass/charge ratio; TL= TissueLyser, N=nymph; M=male; F=female; S=not-engorged, G=blood-engorged.

Supplementary Table S1. Intra and peri-residential prospection of rodent burrows in the Niakhar area.

Villages included	Geographical coordinates	Households investigated	Households positive (%)	Burrows surveyed	Burrows positive (%)	Specimen collected
Diané Bérédji	14°34′ N / 16°18′ W	9	5 (56%)	30	9 (30%)	33
Ndiambour	14°33′ N / 16°20′ W	10	6 (60%)	30	12 (40%)	41
Dione Thiogome	14°35′ N / 16°21′ W	11	4 (36%)	30	10 (33%)	24
Mbakane Néném	14°33′ N / 16°23′ W	6	3 (50%)	30	11 (36%)	34
Patar	14°33′ N / 16°22′ W	9	6 (66%)	30	17 (56%)	48
Wakhaldiam	14°33′ N / 16°24′ W	10	5 (50%)	30	9 (30%)	28
Ngayokheme	14°32′ N / 16°26′ W	34	15 (44%)	75	31 (41%)	121
Ndioulbeth	14°31′ N / 16°22′ W	11	4 (36%)	30	10 (33%)	49
Mbadatte	14°32′ N / 16°21′ W	8	5 (63%)	30	11 (36%)	38
Mback	14°33′ N / 16°20′ W	11	4 (36%)	30	5 (16%)	11
Ndoss Diarraff	14°27′ N / 16°21′ W	8	3 (37%)	30	9 (30%)	22
Ndoss Mbadiokh	14°27′ N / 16°21′ W	9	7 (78%)	30	24 (80%)	109
Ndoss Ndoffene	14°27′ N / 16°21′ W	6	3 (50%)	30	14 (46%)	90
Mbafaye	14°25′ N / 16°24′ W	9	7 (78%)	30	12 (40%)	25
Sagne	14°24′ N / 16°24′ W	8	5 (63%)	30	14 (46%)	47
Godaguéne	14°24′ N / 16°23′ W	9	7 (78%)	30	11 (36%)	24
Mbane	14°22′ N / 16°25′ W	5	3 (60%)	16	10 (33%)	75
**Total**		**173**	**92 (53%)**	**541**	**219 (41%)**	**819**

Supplementary Table S2. Intra and peri-residential prospection of rodent burrows in the Sine-Saloum area.

Villages included	Geographical coordinates	Households investigated	Households positive (%)	Burrows surveyed	Burrows positive (%)	Specimens
Dielmo	13°42′N - 16°24′W	11	2 (18%)	30	4 (13%)	39
Ndiop	13°41′N - 16°23′W	10	4 (40%)	30	6 (20%)	49
Passy	13°41′N - 16°23′W	13	5 (38 %)	30	7 (23%)	96
Touba Nding	13°42′N - 16°25′W	12	7 (58%)	30	9 (30%)	84
Nema Nding	13°42′N - 16°24′W	23	8 (35%)	45	14 (31%)	65
Sabouya	13°43′N -16.23′W	8	1 (13%)	15	1 (7%)	2
Daga Babou	13°45′N - 16°26′W	6	3 (50 %)	19	11 (58%)	106
Keur Aliou Diop	13°42′N - 16°21′W	5	1(20%)	15	1 (7%)	1
Keur Simon Diene	13°45′N - 16°23′W	6	2 (33%)	15	4 (27%)	12
Nema Ba	13°37′N - 16°17′W	8	1 (12%)	15	1 (7%)	2
Medina	13°42′N - 16°24′W	14	0 (0%)	30	0 (0%)	0
Sourou	13.46′N - 16.28′W	8	0 (0%)	15	0 (0%)	0
Bani	13°45′N - 16°28′W	8	0 (0%)	15	0 (0%)	0
Firdawsi	13°45′N - 16°27′W	8	0 (0%)	15	0 (0%)	0
Keur Lahi Socé	13°44′N - 16°25′W	8	0 (0%)	15	0 (0%)	0
**TOTAL**		**148**	**34 (23%)**	**334**	**58 (17%)**	**456**

1

Supplementary Table S3. Identification of *Bartonella* spp. with the closest known species using two different genes from *O. sonrai* ticks in Senegal.

	***GltA* gene detected (200pb)**		***FTSZ* gene (290pb)**	
**Sample**	**GenBank identifications, hosts and accession numbers (similarity, %)**	**Most closely related species fully described; GenBank accession numbers (similarity, %)**	**GenBank identifications, hosts and accession numbers (similarity, %)**	**Most closely related species fully described; GenBank accession numbers (similarity, %)**
Ticks *O. sonrai* 77 OH-FK	Uncultured *Bartonella* sp. *Mastomys erythroleucus* MK892986 (100%)	***Bartonella elizabethae Ctenocephalides felis* MK660539 (98.49%)**	Uncultured Bartonella sp. *M. erythroleucus* MK902922 (99.66%)	***Bartonella mastomydis M. erythroleucus* KY555065 (98.97%)**
Ticks *O. sonrai* 77 SG-FK	*Bartonella* sp. *Cricetomys gambianus* JX428749 (100%)	***Bartonella elizabethae* Homo sapiens blood GQ225710 (98.45%)**	*Bartonella tribocorum Rattus norvegicus* AF467759 (97.60%)	***Bartonella tribocorum* Rattus norvegicus AF467759 (97.60%)**
Ticks *O. sonrai* 86 SG-FK	*Bartonella* sp. *Cricetomys gambianus* JX428749 (100%)		*Bartonella tribocorum* Rattus norvegicus AF467759 (97.60%)	
Ticks *O. sonrai* 48 SG-FK	*Bartonella* sp. Cricetomys gambianus JX428749 (100%)	***Bartonella elizabethae* Homo sapiens blood GQ225710 (98.98%)**	*Bartonella massiliensis Ornithodoros sonrai* 100% HM636443	***Bartonella massiliensis Ornithodoros sonrai* HM636443 (100%)**
Ticks *O. sonrai* 39 OH-FK	*Bartonella* sp. *Cricetomys gambianus* JX428749 (98.49)		*Bartonella massiliensis Ornithodoros sonrai* 100% HM636443	
Ticks *O. sonrai* 19 OH-FK	Uncultured *Bartonella* sp. Xenopsylla cheopis MF443365 (100%)	***Bartonella taylorii***	-	-

Ticks *O. sonrai* 79 OH-FK	Uncultured *Bartonella* sp. Xenopsylla cheopis MF443365 (99.50%)	***Apodemus agrarius*** **AY584853 (94.47%)**	-	
Ticks *O. sonrai* 73 OH-FK	*Bartonella* sp. *Crocidura olivieri* KM233490 (100%)	***Bartonella florencae Crocidura russula*** **HM622142 (97.46%)**	-	-
Ticks *O. sonrai* 107 OH-FK	*Bartonella* sp. *Crocidura olivieri* KM233490 (100%)		-	
